# A cortical biomarker of audibility and processing efficacy in children with single-sided deafness using a cochlear implant

**DOI:** 10.1038/s41598-023-30399-0

**Published:** 2023-03-02

**Authors:** Y. Yaar-Soffer, R. Kaplan-Neeman, T. Greenbom, S. Habiballah, Y. Shapira, Y. Henkin

**Affiliations:** 1 Hearing, Speech, and Language Center, Sheba Medical Center, Tel Hashomer, 52621 Ramat Gan, Israel; 2 Department of Communication Disorders, Sackler Faculty of Medicine, Tel Aviv University, Tel Aviv, Israel; 3 Department of Communication Disorders, Haifa University, Haifa, Israel; 4 Alango Technologies LTD, Tirat Carmel, Israel; 5 Department of Otolaryngology Head and Neck Surgery, Sheba Medical Center, Tel Hashomer, Israel

**Keywords:** Auditory system, Paediatric research

## Abstract

The goals of the current study were to evaluate audibility and cortical speech processing, and to provide insight into binaural processing in children with single-sided deafness (CHwSSD) using a cochlear implant (CI). The P1 potential to acoustically-presented speech stimuli (/m/, /g/, /t/) was recorded during monaural [Normal hearing (NH), CI], and bilateral (BIL, NH + CI) listening conditions within a clinical setting in 22 CHwSSD (mean age at CI/testing 4.7, 5.7 years). Robust P1 potentials were elicited in all children in the NH and BIL conditions. In the CI condition: (1) P1 prevalence was reduced yet was elicited in all but one child to at least one stimulus; (2) P1 latency was prolonged and amplitude was reduced, consequently leading to absence of binaural processing manifestations; (3) Correlation between P1 latency and age at CI/testing was weak and not significant; (4) P1 prevalence for /m/ was reduced and associated with CI manufacturer and duration of CI use. Results indicate that recording CAEPs to speech stimuli in clinical settings is feasible and valuable for the management of CHwSSD. While CAEPs provided evidence for effective audibility, a substantial mismatch in timing and synchrony of early-stage cortical processing between the CI and NH ear remains a barrier for the development of binaural interaction components.

## Introduction

Single Sided Deafness (SSD) is a type of unilateral sensorineural hearing loss (HL), where the degree of HL in the affected ear is classified as severe to profound in the presence of normal or near-normal hearing in the contralateral ear^1^. Accumulating evidence suggests that the absence of binaural hearing results in reduced audibility as well as degraded spatial hearing abilities^2–4^. Consequently, when compared with normal hearing (NH) children, children with SSD (CHwSSD) display reduced speech perception performance in quiet^5,6^ and in noisy conditions^7–9^. Additionally, a growing number of studies indicate poor sound localization with larger variability in CHwSSD compared to their NH peers^2,6,10^.

It is well established that auditory experience during the early stages of life strongly affects the development of the central auditory system^11,12^. Restricted auditory input during early development in CHwSSD has been shown to result in cortical reorganization leaving pathways from the affected ear immature and weakly represented centrally, known as the “aural preference syndrome”^13–16^. Moreover, early auditory deprivation in CHwSSD negatively impacts a wide range of neurodevelopmental functions^17^. These may include aberrant speech and language development^18–22^, poorer neurocognitive function and academic performance^21–26^, increased listening effort^27^, cognitive fatigue^28^, and reduced socio-emotional wellbeing^4,20,29,30^.

Currently, among available management options for SSD, a cochlear implant (CI) is the only intervention that provides direct auditory input to the profoundly deaf ear that could potentially lead to restoration of binaural hearing functions^10,31–36^. Assessing CI benefits in young CHwSSD poses a unique challenge for clinicians due to limited cooperation and difficulty in obtaining auditory thresholds, especially as there is a need to mask the NH ear. Moreover, an advanced level of task understanding is required for assessing binaural hearing abilities (i.e., sound localization, speech perception in noise). Accordingly, most recent studies have provided data on the binaural benefits of a CI only in older CHwSSD. A recent systematic review and meta-analysis reported that in some studies almost 80% of CHwSSD exhibited better speech perception in noise, and improved sound localization skills one to two years after implantation^5^. However, due to the limitations discussed above, only older children could comply and complete the required behavioral tasks.

Evidently, the limitations of behavioral testing in young CHwSSD using a CI highlight the need for objective measures that do not require the child’s active participation and cooperation. Measuring cortical auditory evoked potentials (CAEPs) with a CI provides objective information on audibility, processing efficacy (i.e., timing and synchrony), and cortical plasticity. The P1 potential, the first positive component of the P1–N1–P2 complex, has been used to examine the development and plasticity of the central auditory pathways in children with NH and in those with bilateral deafness using CI^12,37,38^ and may therefore serve as an advantageous biomarker of auditory development in CHwSSD using a CI. It is the only identifiable peak during infancy and early childhood^39^, generated in the primary auditory cortex and thalamus^40^, and decreases in latency with increasing age^41^. Moreover, changes in CAEPs may occur before improvements manifest in behavioral performance^42^. To date, however, only a few studies have explored the use of CAEPs in CHwSSD. In two case reports^43,44^, CAEPs to speech stimuli were recorded pre- and post-implantation in CHwSSD. Despite limited generalization of the findings, these are the first reports which demonstrated that pre-implantation atypical cortical responses from the better ear^43^ and the implanted ear^44^ significantly improved with CI use. Cortical reorganization post-implantation was also supported by Polonenko et al.^45^ and later by Lee et al.^46^, who reported results from cohorts of five and 22 children, respectively. In some of the children, responses to electric pulses from an apical CI electrode resulted in a shift towards normal cortical activity patterns following CI use.

Taken together, the growing body of evidence pointing to the detrimental consequences of SSD cannot be underestimated and has therefore led to continuous growth in the number of CHwSSD receiving a CI. Although current clinical research on CHwSSD using a CI has been conducted in small cohorts, improved performance after implantation has been reported. The significant challenges involved in the clinical care of these children and underdeveloped assessment tools tailored for this unique population, together with the feasibility of measuring CAEPs at a young age, were the main incentives of the current study. Thus, the goals of the study were to evaluate audibility and cortical speech processing, and to provide insight into binaural processing in CHwSSD using a CI. For this purpose, the P1 evoked potential to acoustically-presented speech stimuli was recorded during monaural (NH, CI) and bilateral (BIL; NH + CI) listening conditions within a clinical setting.

## Materials and methods

### Participants

Twenty-two CHwSSD (16 males) who underwent cochlear implantation at the Sheba Medical Center (SMC) between 2019–2021 participated in the study. Demographic and background information are presented in Table 1. All children exhibited NH thresholds in one ear based on auditory brainstem responses to 0.5–4 kHz tone-bursts (≤ 25 dBeHL) or behavioral testing (≤ 20 dBHL), and severe-to-profound sensorineural HL in the contralateral ear (≥ 80 dBeHL/dBHL). The mean age at implantation was 4.68 years (SD = 2.23, range 1.27–8.85), the mean age at testing was 5.75 years (SD = 2.39, range 1.94–9.67), the mean duration of CI use was 12.9 months (SD = 6.63, range 3.85–25.05). All children received continuous auditory rehabilitation including auditory training and used their CI for more than 5 h a day based on data logging and parental report.

**Table 1:** Patients’ demographics (n = 22), presenting the number (and percentage) of participants. *CMV* cytomegalovirus, *CND* cochlear nerve deficiency, *EVA* enlarged vestibular aqueduct, *LCH* Langerhans cell histiocytosis.

Gender	Male 16 (73%), Female 6 (27%)
CI side	Right 11 (50%), Left 11 (50%)
CI Manufacturer	MEDEL 11 (50%), Cochlear 7 (32%), Advanced Bionics 4 (18%)
Hearing Loss Onset	Prelingual 13 (59%), Perilingual 7 (32%), Postlingual 2 (9%)
Etiology	CMV 9 (41%), Meningitis 5 (23%), Unknown 3 (13.5%), CND 2 (9%), EVA 1 (4.5%), Trauma 1 (4.5%), LCH 1 (4.5%)

Ethics approval was obtained from Sheba's Institutional Review Board (reference number: 1534-14 SMC) and included full exemption from informed consent as the utilized methods included routine clinical tests used at the Sheba Medical Center. All tests were performed in accordance with the relevant guidelines and regulations.

### CAEP recordings and stimuli

CAEPs were recorded by means of the Aided Cortical Assessment (ACA) module of the HEARLab™ system^47^. Responses were obtained from three electrodes placed in the following positions: Cz (vertex, active), mastoid contralateral to the CI (reference) and Fpz (ground). Electrode impedances were kept below 5 kOhms. A pre-stimulus baseline of − 200 ms and post-stimulus epoch of 600 ms were pre-determined by the system. Residual noise level was kept below 3.2 μV, and artefact rejection was set at ± 150 μV. CAEPs were elicited by three speech stimuli /m/, /g/, and /t/ with peak intensity in the low, mid, and high frequencies, respectively^47^. The mean duration of all speech stimuli was 79 ms, and the interstimulus interval was 1125 ms. The minimum number of acceptable epochs for responses to each speech signal was 200.

### Procedure

Children sat in a comfortable armchair on their own or on their parent’s lap and watched a silent movie in a sound-treated room. Stimuli were presented at 65 dBSPL from a loudspeaker located 1 m from the child at 0° azimuth in three listening conditions: (1) NH (CI off); (2) Bilateral (BIL; NH + CI); and (3) CI (NH ear masked with white noise). Masking was presented at 65 dBHL via an insert earphone. In order to determine whether adequate masking was provided to the NH ear, five children were tested with and without masking to the NH ear while the CI was turned off. Results indicated that 65 dBHL was sufficient to mask the NH ear, as depicted in Supplementary Fig. S1, showing an absent response with masking to the NH ear and a robust response without masking. A similar amount of contralateral masking was previously reported^48^. Testing lasted between 30–45 min. All children were tested while using their most updated map**.**

### CAEP analysis

HEARLab applies an automatic statistical test (Hotelling's T2) to determine the presence or absence of CAEPs^49^. Ongoing statistical analysis results are shown in the detection p plots, and the detection p-value of < 0.05 indicates the probability that the response is significantly different from noise. The automated statistical detection of cortical responses used in the HEARLab system was reported to be as sensitive as the visual detection of CAEPs by experienced examiners^50^.

In the present study, CAEP presence was determined objectively using the system's automatic statistical test and verified visually by three experienced audiologists/electrophysiologists who determined the presence/ absence of P1. The prevalence, latency, and amplitude of P1 were measured and compared among listening conditions and speech stimuli.

### Speech perception outcomes and parental report

Speech perception with the CI was evaluated via direct audio input (DAI) at a comfortable volume level. Based on the child's performance, the results were classified as one of the following categories: (1) Open-set mono- or bi-syllabic word recognition; (2) Closed-set (4–6 alternatives) bi-syllabic word recognition; and (3) Detection of speech (Ling sounds) and/or environmental sounds. In addition, the results of the Speech, Spatial, and Qualities of Hearing (SSQ) questionnaire^51^ were collected. The questionnaire evaluates hearing measures relying on binaural auditory processing and has been shown to be appropriate for assessing CI benefit in CHwSSD^52^. We utilized an adapted parental version^53^ which includes 23 items that address the following functions: (1) Speech hearing—ability to listen to speech in various hearing contexts; (2) Spatial hearing—localization of sound from different directions, distances, and movement; (3) Qualities of hearing—segregating sounds, identification of sound, clarity, naturalness, and listening effort.

### Statistical methods

ANOVA with repeated measures was used to test the effect of listening condition [NH (CI off), BIL (NH + CI), CI (NH masked)] and stimulus (/m/, /g/, /t/) on P1 latency and amplitude. Post-hoc analysis included Sidak's multiple-comparisons adjustments. Chi-square and two-tailed *t* test were used to test the effect of background variables on the prevalence of P1. Background variables included: CI side, CI manufacturer, HL onset, etiology [grouped into four categories of CMV (n = 9), meningitis (n = 5), inner ear anomaly (n = 2), other (n = 5: 3 unknown, 1 trauma, 1 LCH)], age at implantation, age at testing, duration of CI use, and speech perception category with the CI. Pearson correlation was used to test the association between P1 latency/amplitude and the background variables of age at implantation, age at testing, duration of CI use, and SSQ score. Spearman’s correlation was used to test the association between P1 latency/amplitude and speech perception category. Significance was defined at p < 0.05. Effect size measures were Partial Eta Squared (η_p_^2^) for ANOVA, Cohen’s d for *t* tests, and Cramer’s V for chi-square analysis. All analyses were performed with the SPSS statistical package^54^.

## Results

### P1 prevalence

**Figure 1:**
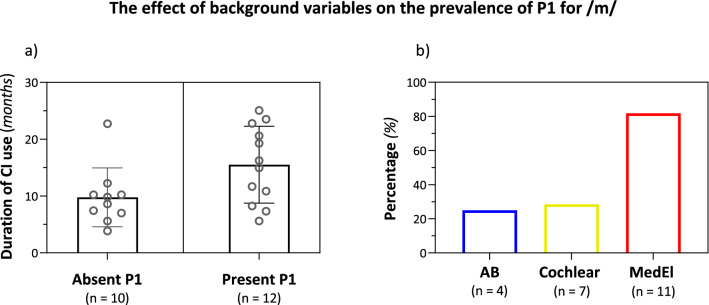
P1 prevalence for /m/ demonstrated for the background variables of (**a**) duration of CI use (months)—showing individual data points and means (± SD), indicating a significant difference between absent and present P1 (mean of 9.77 vs. 15.95 months, respectively) (*p* = 0.04). (**b**) CI manufacturer—showing P1 prevalence for each manufacturer, indicating a significantly higher prevalence in children implanted with the Medel device (81.8%) compared to Cochlear (28.5%) and Advanced Bionics (AB) (25%) (*p* = 0.037).

The overall prevalence of P1 elicited in the NH, BIL, and CI listening conditions was 100%, 100%, and 78%, respectively. P1 prevalence for /m/, /g/, and /t/ in the CI condition was 52%, 95%, and 85%, respectively. Duration of CI use as well as CI manufacturer significantly affected the prevalence of P1 for /m/ [*t*(20) = 2.19, *p* = 0.04, Cohen’s *d* = 0.94; *X*^2^(2) = 6.6, *p* = 0.037, Cramer’s *V* = 0.55]. As shown in Fig. 1a, P1 for /m/ was absent in 10 children that used their CI for a mean of 9.77 months and was present in 12 children that used their CI for a mean of 15.95 months. Figure 1b shows the prevalence of P1 for /m/ according to CI manufacturer indicating that prevalence was significantly higher in children implanted with the Medel device (81.8%) compared to Cochlear (28.5%) and Advanced Bionics (AB) (25%). Background variables that did not significantly affect P1 prevalence for /m/ were HL onset (*p* = 0.148), etiology (*p* = 0.881), CI side (*p* = 0.392), speech perception category (*p* = 0.067), age at implantation (*p* = 0.278), and age at testing (*p* = 0.135).

### The effect of listening condition

**Figure 2:**
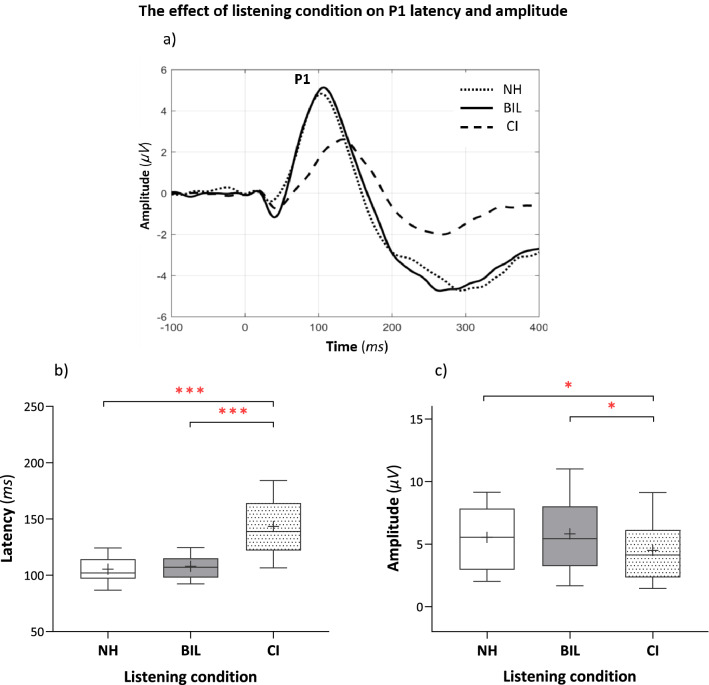
The main effect of listening condition [NH, BIL (NH + CI), CI (NH masked)] on P1 latency and amplitude. (**a**) Grand average CAEP waveforms elicited in the three listening conditions, depicting the significant main effect of listening condition on P1 latency (*p* < 0.001) and amplitude (*p* = 0.007); (**b**,**c**) Boxplots illustrating pairwise comparisons. The bottom and top of the box represent the 25th to 75th percentiles (respectively), the middle solid line represents the median, the + symbol represents the mean, and the whiskers extend between the 10th and 90th percentiles. P1 latency was longer and amplitude was reduced in the CI vs. the NH condition (*p* < 0.001, *p* = 0.026, respectively) and to the BIL condition (*p* < 0.001, *p* = 0.021, respectively). Differences between the latencies and amplitudes in the NH and BIL conditions were not significant (*p* = 0.853, *p* = 1.00, respectively). *p < 0.05, ***p < 0.001.

Grand average CAEP waveforms elicited in the three listening conditions are presented in Fig. 2a. P1 was robust and similar in morphology in the three listening conditions. Group mean data of P1 latency and amplitude are presented in Fig. 2b,c and statistical analysis results are presented in Table 2. A significant main effect of listening condition on P1 latency and amplitude was found. As seen in Fig. 2b,c, P1 latency was on average 40.9 ms longer and amplitude was 1.55 µV smaller in the CI compared to the NH condition. Similarly, P1 latency was on average 39.41 ms longer and amplitude was 1.54 µV smaller in the CI compared to the BIL condition. No significant differences between the latencies and amplitudes in the NH and BIL conditions were evident.

**Table 2:** Main effects and pairwise comparisons of listening condition, stimulus, and listening condition by stimulus interactions on P1 latency and amplitude. Post-hoc analysis included Sidak's multiple-comparisons adjustments. *df d*egrees of freedom, *η*_*p*_^*2*^ effect size partial Eta squared, p-value—bold indicates statistical significance (*p* < 0.05).

	F (*df*)	p-value	η_p_^2^
**Effect of listening condition**			
P1 latency			
Main effect	57.21 (1.22, 25.71)	**< 0.001**	0.73
Paired comparisons			
CI-NH		**< 0.001**	
CI-BIL		**< 0.001**	
NH-BIL		0.853	
P1 amplitude			
Main effect	52.42 (2, 42)	**0.007**	0.21
Paired comparisons			
CI-NH		**0.026**	
CI-BIL		**0.021**	
NH-BIL		1.00	
**Effect of stimulus**			
P1 latency			
Main effect	10.42 (1.55, 32.48)	**0.001**	0.33
Paired comparisons			
/m/-/t/		**0.009**	
/m/-/g/		**0.001**	
/t/-/g/		0.932	
P1 amplitude			
Main effect	2.41 (1.47, 30.85)	0.120	0.10
**Listening condition × Stimulus interaction**			
P1 latency	0.69 (2.28, 47.95)	0.524	0.03
P1 amplitude	1.01 (4, 71.43)	0.405	0.04

### The effect of stimulus

**Figure 3:**
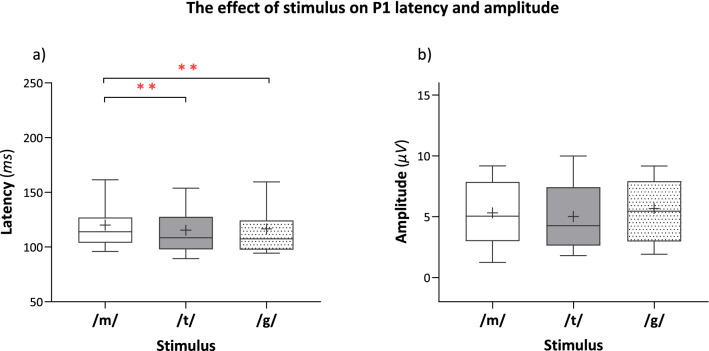
The main effect of stimulus (/m/, /g/, /t/) on P1 latency and amplitude presented by boxplots illustrating pairwise comparisons. The bottom and top of the box represent the 25th to 75th percentiles (respectively), the middle solid line represents the median, the + symbol represents the mean, and the whiskers extend between the 10th and 90th percentiles. (**a**) P1 latencies elicited by /m/ were significantly prolonged compared to the latencies elicited by /t/ (*p* = 0.009) and by /g/ (*p* = 0.001). P1 latency to /t/ and /g/ did not significantly differ (*p* = 0.932). (**b**) The main effect of the stimulus on P1 amplitude was not significant (*p* = 0.12). **p < 0.01.

**Figure 4:**
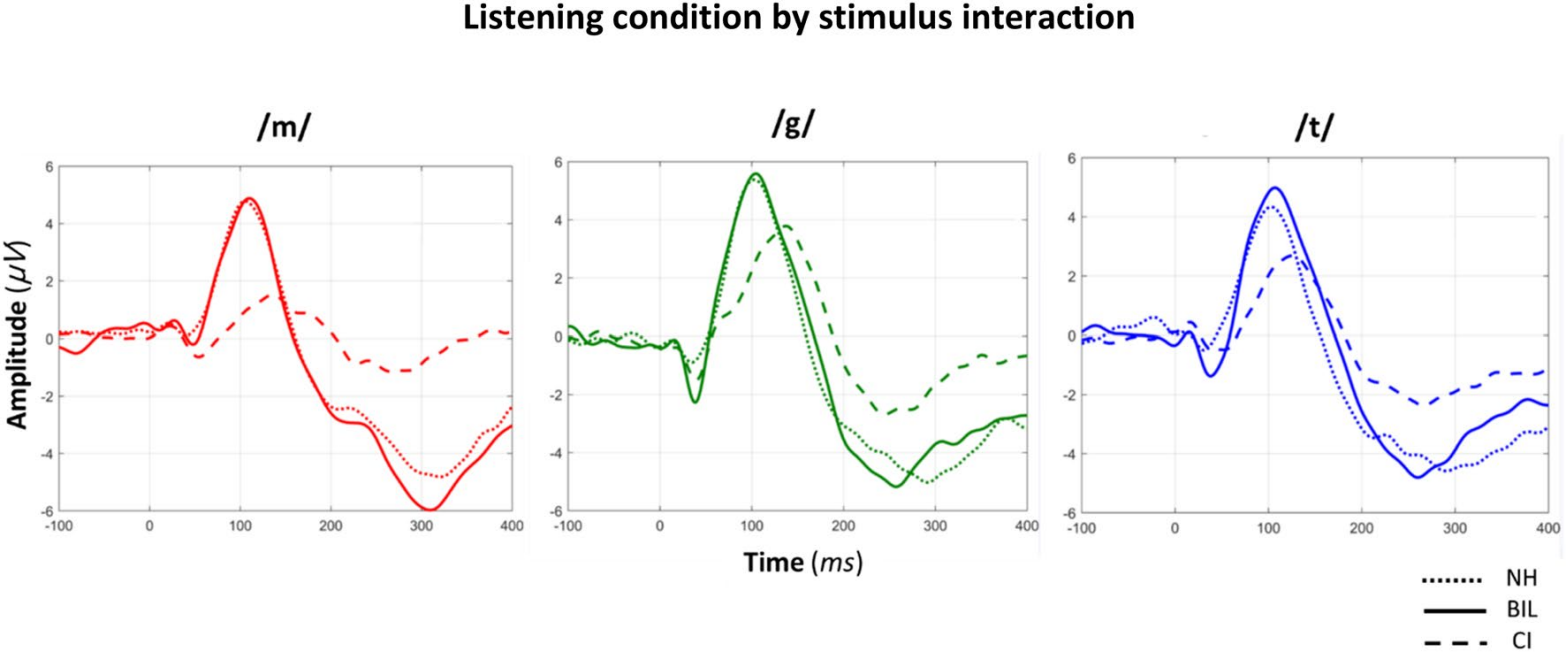
Grand average CAEP waveforms elicited by each stimulus (/m/, /g/, /t/) in the three listening conditions [NH, BIL (NH + CI), CI (NH masked)], depicting the non-significant listening condition by stimulus interaction for P1 latency (*p* = 0.524) and amplitude (*p* = 0.405).

Group mean data of P1 latency and amplitude for each stimulus (/m/, /g/, /t/) are presented in Fig. 3a,b and statistical analysis results are presented in Table 2. A Significant main effect of stimulus on P1 latency was found. As seen in Fig. 3a,b, P1 latencies elicited by /m/ were on average 10 ms longer compared to the latencies elicited by /t/ and 8.84 ms longer compared to the latencies elicited by /g/. P1 latency to /t/ and /g/ did not significantly differ (pairwise comparisons). The main effect of the stimulus on P1 amplitude was not significant. Finally, the stimulus by listening condition interaction was not significant for both latency and amplitude, as shown in Fig. 4 which depicts grand average CAEP waveforms for each stimulus separately in the three listening conditions.

### Correlations between P1 latency/amplitude and background variables

**Figure 5:**
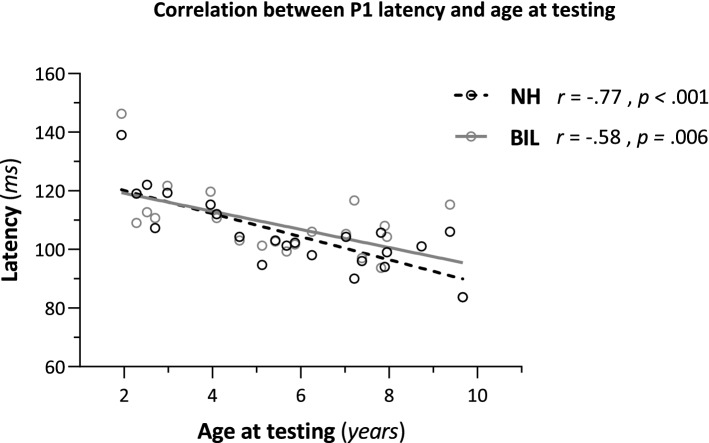
Correlations between P1 latency and age at testing in the NH (black dashed) and BIL (CI + NH) (grey solid) listening conditions. Individual data points represent mean latency for all three stimuli. Strong and moderate negative correlations were found for NH [*r*(20) = − 0.77, *p* < 0.001] and BIL [*r*(19) = − 0.58, *p* = 0.006] listening conditions, respectively.

Figure 5 presents the correlations between age at testing and P1 latency in the NH and BIL listening conditions. Strong and moderate (respectively) negative correlations were found indicating latency shortening with increasing age [NH: *r*(20) = − 0.77, *p* < 0.001; BIL: *r*(19) = − 0.58, *p* = 0.006]. The correlations between age at testing and P1 amplitude in the NH and BIL listening conditions were weak and not significant [NH: *r*(20) = − 0.03, *p* = 0.912; BIL: *r*(19) = − 0.03, *p* = 0.909].

In the CI listening condition, the correlations between P1 latency/amplitude and age at implantation, age at testing and duration of CI use were weak and not significant [age at implantation: latency—*r*(19) = − 0.04, *p* = 0.851, amplitude—*r*(19) = − 0.06, *p* = 0.795; age at testing: latency—r(19) = 0.13, *p* = 0.563, amplitude—*r*(19) = − 0.09, *p* = 0.710; duration of CI use: latency—*r*(19) = − 0.39, *p* = 0.078, amplitude—*r*(19) = − 0.12, *p* = 0.594].

### Speech perception outcomes and SSQ parental report

Results indicated that open-set, closed-set, and detection-only speech perception abilities were evident in 50%, 30%, and 20% of the children, respectively. Correlations between speech perception and P1 latency/amplitude were weak and not significant [latency—*r*(19) = − 0.21, *p* = 0.357, amplitude—*r*(19) = − 0.03, *p* = 0.88].

The SSQ questionnaire was completed by 16/22 parents. Mean SSQ score was 7.2 (SD = 1.39, range 4.1–9). Correlations between SSQ score and P1 latency and amplitude were weak and not significant [latency—*r*(14) = − 0.29, *p* = 0.269, amplitude—*r*(14) = − 0.29, *p* = 0.274].

## Discussion

As the number of CHwSSD receiving a CI continues to grow, CI programs are facing significant challenges in obtaining information regarding adaptation and acquirement of auditory function of the implanted ear, as well as development of binaural hearing abilities. The main goal of the present study was to evaluate audibility and cortical speech processing efficacy in young CHwSSD using a CI by means of an objective measure, i.e. CAEP, that does not require active cooperation. An additional goal was to explore whether CAEP can be used as a clinical tool for assessing binaural hearing. The P1 potential was elicited in three listening conditions: NH (CI off), BIL (CI + NH), and CI (NH masked) to three speech stimuli (/m/, /g/, and /t/). To the best of our knowledge, this is the first study in which cortical potentials were recorded to speech stimuli presented acoustically, within a clinical setting, from a relatively large cohort of CHwSSD using a CI. Moreover, current data provide novel, within-subject comparisons between cortical activity while hearing with the CI ear, with the NH ear, and with both ears.

### Hearing with the CI ear vs. the NH ear

Results of the current study indicated that all children exhibited age-appropriate CAEP morphology that included a robust P1 potential to the three speech stimuli in the NH ear and BIL listening conditions. In the CI ear: (1) P1 morphology was similar to that of the NH ear; (2) P1 prevalence was reduced yet was elicited in all but one child, to at least one of the three speech stimuli, indicating effective audibility at typical conversational levels (65 dBSPL); (3) P1 latency was significantly prolonged and amplitude was reduced compared to P1 of the NH ear, signifying substantial mismatch in timing and synchrony of early-stage cortical processing; and (4) The correlation between P1 latency and age at testing was weak and not significant, whereas the NH ear showed strong and significant correlation indicating the expected shortening of P1 latency with increasing age.

The finding that the CI ear and NH ear showed similar P1 morphology may be explained by the well-established notion that auditory stimulation by means of a CI results in morphological and functional changes due to the increased number of responsive neurons to sound, expanded dendritic branching, enhanced myelination, and restored synaptic connections^55^. The very few published studies in the literature on cortical processing efficacy in CHwSSD focused on cortical reorganization. In a single case report study, Sharma et al.^44^ reported on a child with post-lingual SSD who was implanted at the age of 9.8 years. CAEPs elicited by the syllable /ba/ presented from a loudspeaker at 65 dBHL, while plugging and muffing the NH ear, showed significant changes post-CI. After 3 months of CI use P1 was evident in the implanted ear while the age-appropriate P1-N1-P2 complex was evident in the NH ear. By 14 months of CI use the implanted ear showed the expected P1–N1–P2 complex. Two additional studies explored brain activation patterns in CHwSSD post-CI by means of acoustic and electric stimulation of the NH and CI ear, respectively^45,46^. While aural preference, i.e. enhanced representation of the better hearing ear in the auditory cortices^13^, was evident at initial CI stimulation, increasing CI use resulted in decreased cortical response to stimulation of the NH ear, signifying reduced reliance on the NH ear and strengthening of pathways from the implanted ear. The authors postulated, however, that in some children aural preference is difficult to reverse as audibility is higher in the NH vs. CI ear^46^.

Because CAEP can be elicited by longer duration stimuli, their presence can confirm cortical detection of speech sounds (audibility) at conversational levels^56^. In children, however, the association between the presence of CAEPs to speech sounds and audibility (behavioral detection) has not been investigated directly. In children with bilateral hearing loss using hearing aids P1 to the speech sounds /m/, /g/, and /t/presented at normal conversational levels (55–75 dBSPL) correlated with results of the Parents' Evaluation of Aural/Oral Performance of Children (PEACH) questionnaire^57^. Data from NH and hearing-impaired adults recorded with the HearLab AEP system, also used in the current study, indicated that CAEPs presence occurred within 10 dB of the behavioral thresholds^58,59^. Taken together, previous findings and our data provide support to the notion that in CHwSSD using a CI, CAEPs may serve as an objective clinical measure of audibility. Clinically, the utilization of CAEP may delay the challenging need to obtain behavioral thresholds in the presence of a NH ear in infants and young children. Importantly, information regarding audibility is essential for auditory training, CI programming, and for potentially acquiring binaural hearing abilities.

A novel finding of the present study is the significant P1 latency and amplitude differences between the CI ear and the NH ear (mean difference 40.9 ms and 1.55 µV, respectively). This finding indicates substantial delay and reduced synchrony during initial cortical processing of acoustically-presented speech stimuli in the CI ear, taking place at the primary auditory cortex. Auditory deprivation and aberrant cortical organization also reported in kittens with congenital SSD, presumably underlie this finding^14,60^. The timing mismatch between the NH and CI ear has substantial negative implications for processing binaural timing and level cues required for developing binaural hearing abilities such as sound localization and speech understanding in noise^61^ that may resolve due to plasticity and re-organization with increasing CI use^45,46^. Insight regarding the differences in auditory processing between the NH and CI ears can be obtained from CAEPs of post-lingual adult SSD patients using a CI^62–64^. While actively processing sinusoidal tones^63^, syllables^64^, and words^62^, response times were significantly longer in the CI vs. NH ear. Slower responses were in accord with enhanced listening effort reported while listening with the CI and with prolonged latencies of N1 and P3 and reduced amplitudes of N1 and P2 in the CI vs. NH ear. These differences were explained by the degraded auditory signal provided by the CI together with limited capacity of the auditory cortex to adapt to the CI signal^64^. In contrast Wedekind et al.^65^ reported similar N1 and P2 latencies in a NH vs. CI (NH masked) listening condition using similar methodology to the one used in the present study (/m/, /g/, /t/, and /s/ stimuli presented acoustically by means of the HearLab system). The authors suggested that reactivation of auditory pathways in adult post-lingual SSD patients can take place even after many years of deprivation, and explain restoration of behavioral binaural hearing abilities. Clearly, the inconsistency among previously published reports may stem from differences in background characteristics of the studied cohorts and implemented methodologies, and thus requires further investigation.

An additional notable difference between the CI ear and the NH ear manifested in the differential developmental trajectory of P1 latency as a function of increasing age at testing from 1.9 to 9.7 years. The NH ear exhibited the expected, well-established shortening of P1 latency found in children with NH with increasing chronological age (e.g., Sharma et al.^12^; data from 136 NH children). This finding solidifies our results and supports the use of CAEP as a biomarker of early cortical speech processing. The latency of P1 of the CI ear, however, was not correlated with increasing age at testing and should be interpreted cautiously as children differed, to some extent, in age at implantation and duration of CI use.

### Hearing with two ears (NH + CI)

One of the goals of the present study was to explore whether the P1 potential could serve as a biomarker of cortical binaural processing. A wealth of electrophysiological literature in NH listeners provides evidence for binaural interaction components (BICs) that occur along the auditory pathways and manifest in the ABR^66^, MLR/LLR^67^, and ERP^68^. BICs are derived responses that reflect binaurally-evoked neural events and are thought to be associated with sound localization/lateralization, based on their dependence on interaural time and intensity differences^69^. The basic principle of BICs is that information presented monaurally to the right and left ears interact, and consequently binaural presentation results in a deviation from the sum of the monaural outputs^66^. Typically, BICs are derived by subtracting the binaural response from the sum of monaural responses. In the current study, however, BIC could not be derived due to the substantial latency asymmetry between the NH and CI ear. Another method to obtain information regarding binaural processing is to compare evoked potentials to bilateral vs. monaural stimulation, where greater binaural amplitudes are expected^70^. In the current study, the remarkably similar P1 amplitude and latency in the BIL and NH listening conditions support aural preference of the NH ear, and presumably limited contribution of the CI ear to the binaural response. Previous studies suggest that mismatched time, frequency, and level of inputs reaching the binaural neurons from the NH and CI ear may explain the poor binaural abilities of SSD patients using a CI^71^. Moreover, the authors concluded that attempts to compensate for the substantial mismatch by means of programming the CI, are ambitious, and that even in an ideal case, binaural abilities of an SSD patient using a CI will not surpass that of a good bilateral CI patient. We therefore assume that with increasing CI use in our studied cohort, maturation of the deprived auditory pathways will take place, asymmetry between the ears will decrease, and consequently BICs may emerge. It should be kept in mind that current limitations of CI devices continue to pose significant drawbacks for achieving true binaural processing.

### The effect of stimulus on early-stage cortical processing with the CI and NH ear

One of the advantages of the current study was the use of three stimuli /m/, /g/, and /t/ presented acoustically, with energy peaks in the low, mid and high frequencies (respectively) covering a wide range of the speech spectrum. Furthermore, all stimuli duration was 79 ms ruling out the possibility that differences in duration may have affected the characteristics of the cortical response^72^.

P1 elicited by /m/ was prolonged compared to P1 elicited by /g/ (8.8 ms) and /t/ (10 ms). Nonetheless, the stimulus by listening condition interaction was not significant. Previous studies indicated prolonged P1 latency to /m/ compared to /t/ in NH infants^39,57^, in children with pre-lingual bilateral deafness using a unilateral CI (Kosaner et al., 2018^73^; /m/ vs. /g/, /t/), and in post-lingual SSD adult CI users (Wedekind et al.^65^; /m/ vs. /g/, /t/, /s/). Interestingly, in children with bilateral pre-lingual deafness P1 latency for /m/ decreased with increasing CI use from one to > 6 months of use, yet P1 latency for /m/ remained longer when compared to /g/ and /t/^73^. This consistent finding may be explained by the notion that stop consonants like /t/ have more energy at stimulus onset compared to non-stop (bilabial) consonants like /m/, as suggested in a magnetoencephalographic study showing longer latencies to words with initial non-stop consonants vs. stop consonants in five adult NH listeners^74^.

Additionally, while P1 was elicited by all stimuli in the NH and BIL listening conditions, in the CI condition the prevalence of P1 elicited by /m/ (52%) was reduced compared to /g/ (95%) and /t/ (85%). Further analysis revealed that the background variables CI manufacturer and duration of CI use, were associated with P1 prevalence. P1 for /m/ was present in the vast majority of Medel users (82%) and only in 25% and 28% of AB and Cochlear users. A similar prevalence of P1 for /m/ was reported by Kosaner et al.^73^, who tested a group of children implanted with the Medel CI who used the same coding strategy as the one used by our cohort, for over 6 months. The finding of higher accessibility to the low-frequency content /m/ stimulus in Medel CI users is innovative, and to the best of our knowledge has not been previously reported. We suggest this result reflects differences in CI coding strategies among CI manufacturers. Generally, all speech coding strategies present several limitations, such as electrical stimulation spread of excitation, imprecise coding of temporal envelope modulations, and limited number of channels for low frequency coding^75^. These might result in poor access to low frequency stimuli, such as the /m/ sound, with a frequency range of 200–500 Hz^47^. The Medel coding strategy, however, specifically the Fine Structure strategy (FS4) in which all our Medel users were programmed with, provides envelope of the bandpass filter outputs together with fine structure cues. Typically, at the four apical electrodes, in addition to envelope information conveyed by all three manufacturers, low frequency information is delivered by bursts of stimulation, triggered by positive zero-crossings in the bandpass-filtered waveforms^75^. Thus, additional activation at the low frequency bands using the FS4 strategy may have increased accessibility and consequently led to the higher prevalence of P1 for /m/ in Medel users compared to Cochlear and AB users.

Another background factor that was associated with P1 prevalence for /m/ is the duration of CI use. P1 was present in 54.5% of the children that used their CI for a mean of 16 months, but was absent in the remaining 45.5% that used their CI for a mean of 10 months. Similarly, in children with bilateral deafness using a unilateral Medel device the prevalence of P1 for /m/ increased from one to > 6 months and was approximately 30% and 80%, respectively^73^. As all children in our cohort received auditory training and were consistently using their CI, we suggest that the emergence of P1 for /m/ follows a differential maturational trajectory. These results were taken into account in the clinical management of the children with absent P1 for /m/ and they were scheduled for a programming session in an attempt to improve audibility in the low frequency range (currently under study). Finally, the additional background factors of HL onset, etiology, CI side, speech perception category, and age at testing did not affect P1 prevalence for /m/.

### The association between CAEP and outcome measures

In the current study a gross speech perception outcome measure was utilized (categorical performance level) due to young age and limited cooperation in behavioral evaluation, which did not correlate with the electrophysiological results. Nonetheless, half of the studied cohort exhibited open-set speech recognition, and parental reports (SSQ scores) were high^76,77^. The positive parental feedback on their child's auditory function post-CI may have been affected, to some extent, by their decision to pursue cochlear implantation, a non-trivial new intervention for CHwSSD. Finally, the behavioral performance, parental reports, and electrophysiological data provide support for the favorable outcomes of cochlear implantation in CHwSSD, a topic that will continue to be a focus of interest for clinicians and scientists.

In conclusion, recording CAEPs to acoustically-presented stimuli covering a wide range of the speech spectrum, in a clinical setting, is feasible and clinically valuable for the management and rehabilitation of CHwSSD. While CAEPs provided evidence for effective audibility in the CI ear, substantial mismatch in the timing and synchrony of early-stage cortical processing between the CI and NH ear remains a barrier for the development of cortical binaural interactions. The current results together with the opportunity to develop binaural hearing with continuous CI use and auditory training, provide support to cochlear implantation for CHwSSD.

## Supplementary Information

Supplementary Figure S1.

## Data Availability

The datasets used and/or analyzed during the current study are available from the corresponding author on reasonable request.
