# Supplementary material for: A cortical biomarker of audibility and processing efficacy in children with single-sided deafness using a cochlear implant
Source: Sci Rep. 2023 Mar 2;13:3533. doi: 10.1038/s41598-023-30399-0 (PMC9981742; doi:10.1038/s41598-023-30399-0)
Supplement: Supplementary file 1 — Supplementary Figure S1. [file 41598_2023_30399_MOESM1_ESM.docx]

**Supplementary material**

**a) NH ear with masking**


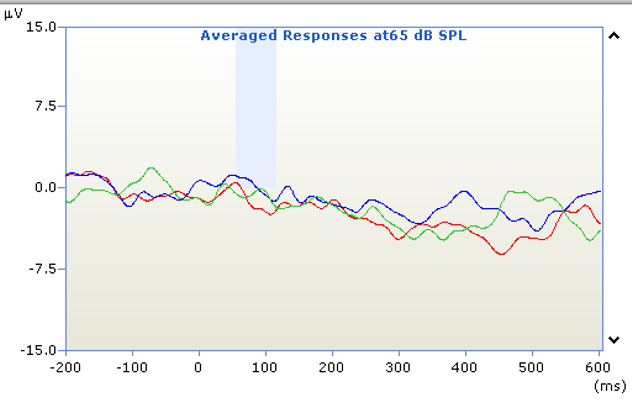

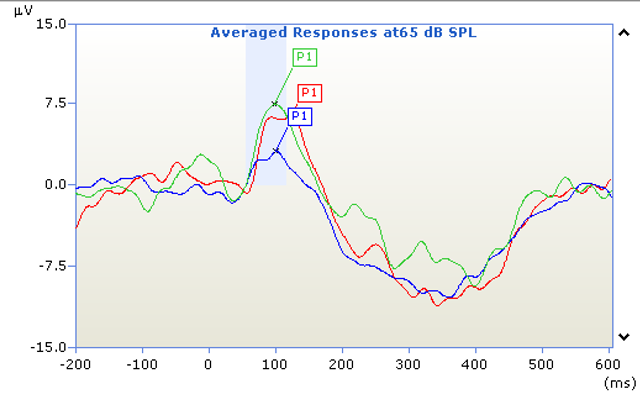


**b) NH ear without masking**

**Supplementary Figure S1.**

‎ **Figure S1**. The figure presents representative CAEP waveforms elicited by /m/ (red), /g/ (green), and /t/ (blue). To determine whether adequate masking was provided to the NH ear, five children ‎were presented with all speech stimuli at 65 dBSPL from a loudspeaker located 1 meter from the child at 0° ‎azimuth. Children were tested in the following listening conditions while the CI was turned off: a) With white noise masking at 65 dBHL presented via an insert earphone to the NH ear, resulting in absent responses. b) Without masking, resulting in robust responses from the NH ear for all speech stimuli. The findings indicated that 65 dBHL was sufficient to mask the NH ear.
